# Editorial Comment: Systematic Literature Review and Meta-Analysis of Sacral Neuromodulation (SNM) in Patients with Neurogenic Lower Urinary Tract Dysfunction (nLUTD): Over 20 Years’ Experience and Future Directions

**DOI:** 10.1590/S1677-5538.IBJU.2021.06.02

**Published:** 2021-10-01

**Authors:** Marcio Augusto Averbeck

**Affiliations:** 1 Hospital Moinhos de Vento Unidade de Videourodinâmica Porto AlegreRS Brasil Chefe de Neuro-Urologia, Unidade de Videourodinâmica, Hospital Moinhos de Vento. Porto Alegre, RS, Brasil

Arndt van Ophoven 1, Stefan Engelberg 2, Helen Lilley 3, Karl-Dietrich Sievert 4, 5, 6

Adv Ther. 2021 Apr;38(4):1987-2006.

## COMMENT

Sacral neuromodulation (SNM) is an established third-line treatment for idiopathic lower urinary tract dysfunctions (LUTD) in patients who failed conservative therapies, such as behavioral and pharmacological strategies (1). Most studies on SNM focused on the role of this minimally invasive treatment in patients presenting idiopathic overactive bladder (iOAB), chronic non-obstructive urinary retention and chronic pelvic pain. However, there is increasing evidence supporting the use of SNM for patients with adult neurogenic lower urinary tract dysfunction (ANLUTD). According to the International Continence Society (ICS), neurogenic overactive bladder (nOAB) is characterized by ‘urgency, with or without urgency urinary incontinence, usually with increased daytime frequency and nocturia in the setting of a clinically relevant neurologic disorder with at least partially preserved sensation (2). Neurogenic OAB is a common presentation of several neurologic diseases, including CNS lesions (stroke, Parkinson’s disease, tumors, etc.) and spinal cord lesions. Studies on SNM for patients with neurological diseases tend to follow the same criteria used for patients with idiopathic LUTD (3).

Van Ophoven et al. have performed a systematic literature review and meta-analysis of studies reporting the safety and effectiveness of SNM in patients with ANLUTD (neurogenic detrusor overactivity, non-obstructive urinary retention, or a combination of both). Forty-seven studies were included in the systematic literature review. Twenty-one studies comprising a total of 887 patients were included in the meta-analysis of test SNM. The pooled success rate of SNM test stimulation was 66.2% (95% CI 56.9–74.4). Depending on neurogenic conditions test success rates varied greatly. Twenty-four studies with a total of 428 patients were included in the meta-analysis of permanent SNM. The success rate of pooled permanent SNM was 84.2% (95% CI 77.8–89.0). Among the identified studies, the most common adverse events (AEs) were loss of effectiveness, infection, pain at implant site, and lead migration with AE rates of 4.7%, 3.6%, 3.2%, and 3.2%, respectively.

These outcomes are consistent with the meta-analysis published by Kessler et al. (4) in 2010, which demonstrated a pooled success rate of 68% for the test phase and of 92% for permanent SNM implant, with a mean follow-up of 26 mo.

Although SNM is a promising treatment for neuro-urological patients, available studies on SNM for neurogenic LUTD are based on small sample sizes and heterogeneous populations, which are incompletely characterized in terms of severity of neurologic impairment, lacking standardized definitions of success and follow-up. On the other hand, the need for serial imaging of the central nervous system (CNS) in selected neuro-urological patients was a barrier to the dissemination of this method, since, until recently, there were no MRI-compatible devices. Newer technologies, such as rechargeable and full-body MRI-compatible devices, may help increase the level of evidence in the near future.
